# Comprehensive Treatment of Total Arch Replacement in a Patient With Liver Cirrhosis: A Case Report

**DOI:** 10.7759/cureus.60365

**Published:** 2024-05-15

**Authors:** Hiromasa Nakamura, Yujiro Miura, Keisuke Yoshida, Ren Saito, Kazumasa Orihashi

**Affiliations:** 1 Cardiovascular Surgery, Kochi Medical University Hospital, Nankoku, JPN; 2 Cardiovascular Disease, Kochi Medical University Hospital, Nankoku, JPN; 3 Liaison Healthcare Engineering, Kochi Medical University Hospital, Nankoku, JPN

**Keywords:** pre-operative management, model for end-stage liver disease score, partial splenic embolization, liver cirrhosis, total arch replacement

## Abstract

A 78-year-old woman with liver cirrhosis due to chronic hepatitis C visited our department for treatment of a thoracic aortic aneurysm. Her Child-Pugh classification was class A, and her model for end-stage liver (MELD) disease score was 8. As she also had thrombocytopenia associated with splenomegaly and esophageal varices, endoscopic injection sclerotherapy and partial splenic embolization were performed before total arch replacement surgery for treating esophageal varices to reduce the bleeding risk during transesophageal echocardiography and for thrombocytopenia, respectively. After endoscopic injection sclerotherapy and partial splenic embolization, the platelet count increased; hence, total arch replacement surgery was performed. By combining partial splenic embolization and endoscopic injection sclerotherapy, we were able to safely perform transesophageal echocardiography and total arch replacement surgery in the perioperative period.

## Introduction

In heart surgery, liver cirrhosis (LC) carries risks that are not reflected by JapanSCORE2 (https://play.google.com/store/apps/details?id=org.jcvsd.JapanSCORE&hl=en&gl=US) or EuroSCORE II (https://www.euroscore.org/index.php?id=17); hence, careful perioperative management is necessary. Bleeding associated with coagulation abnormalities and esophageal varices, which are relative contraindications to transesophageal echocardiography (TEE), are important considerations. Partial splenic embolization (PSE), which is an established treatment for pancytopenia secondary to splenomegaly associated with LC, may potentially decrease portal vein pressure in patients with portal hypertension [1].

Herein, we report a patient with LC who had a model for end-stage liver disease (MELD) score of 8 and class A Child-Pugh classification who underwent total arch replacement (TAR) following PSE for LC-associated complications.

## Case presentation

**Figure 1:**
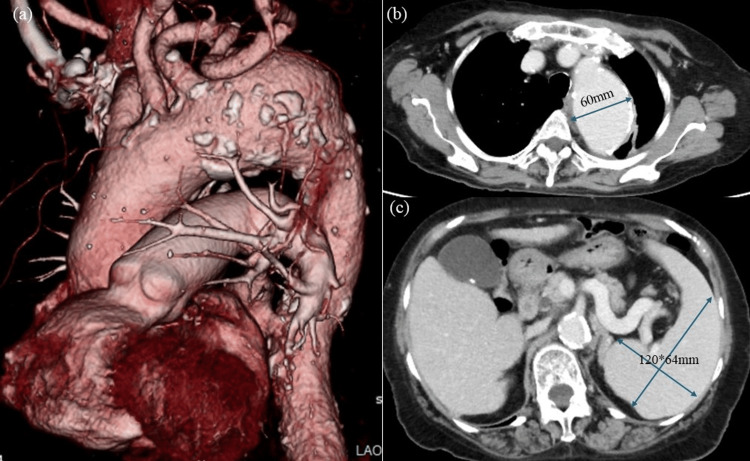
Preoperative computed tomography images (a) and (b) show the distal arch aneurysm with a diameter of 60 mm. The left subclavian artery was derived from the aneurysm. (c) shows splenomegaly (120 x 64 mm).

The patient was a 78-year-old woman who was followed up for hypertension and liver dysfunction. On chest computed tomography (CT), a thoracic aortic aneurysm was incidentally detected, prompting a referral to our department for surgery. Physical examination revealed splenomegaly, thrombocytopenia, and esophageal varices, which were associated with chronic hepatitis C. Blood testing revealed the following results: total bilirubin (T-Bil): 0.8 mg/dL, cholinesterase (ChE): 123 U/L, albumin (Alb): 3.9 g/dL, creatinine (Cre): 0.72 mg/dL, hemoglobin: 12.7 g/dL, platelet count: 9.3 (x10,000/µL), and international normalized ratio (INR): 1.11. CT showed a distal arch aortic aneurysm measuring 60 mm, and the left subclavian artery was derived from the aneurysm. Splenomegaly (120 x 64 mm) was also observed (Figure 1). Abdominal echography showed a small amount of ascites, but the liver surface was smooth, and its margins were sharp. Endoscopy showed F2 esophageal varices with the following characteristics: location, L1; form, F2-3; red color (RC), sign 0; telangiectasia, Te1. Coronary angiography showed no significant coronary artery stenosis. Regarding LC, the patient’s Child-Pugh classification was class A (no encephalopathy: 1 point; amount of ascites: 2 points; Alb 4.4 g/dL: 1 point; T-Bil 1.1 mg/dL: 1 point; prothrombin time (PT) activity: 78.3%: 1 point; total 6 points), and her MELD score was 8.

We planned that the patient was first treated for thrombocytopenia associated with splenomegaly followed by TAR for thoracic aortic aneurysm. Endoscopic injection sclerotherapy (EIS) was initially performed for esophageal varices to reduce the risk of bleeding during TEE, which is essential for intraoperative diagnostic monitoring and navigation, followed by PSE for thrombocytopenia. Thereafter, TAR was performed to definitively treat the thoracic aortic aneurysm.

**Figure 2:**
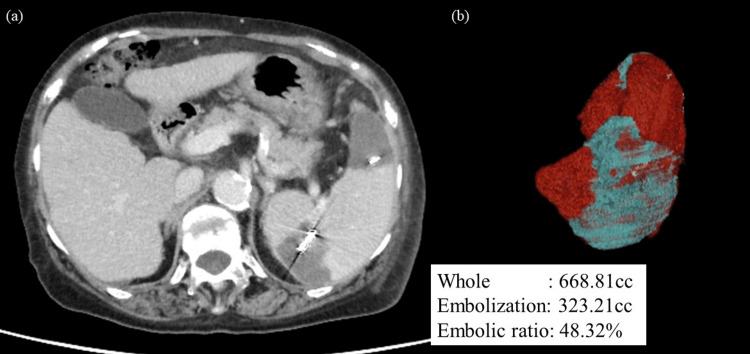
Computed tomography images showing changes in the spleen after partial splenic embolization (a) shows the effects of partial splenic embolization. The upper and middle portions of the spleen were embolized. (b) shows the spleen embolization rate. Approximately 48% of the spleen was embolized.

**Figure 3:**
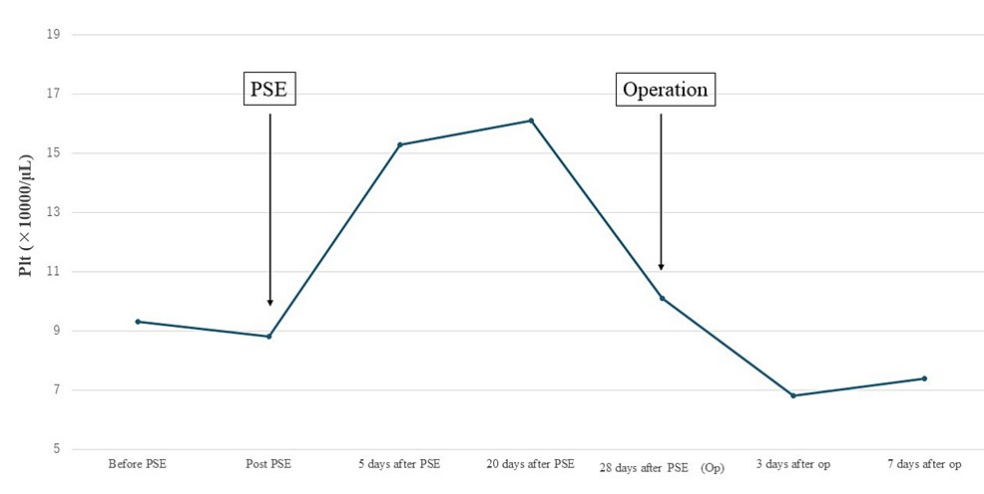
Changes in platelet count during the perioperative period Before PSE, the platelet count was 93,000, which increased to 150,000 on the fifth day and 100,000 on the twentieth day after PSE. On the twenty-eighth day after PSE (day of surgery), the platelet count decreased to 100,000. PSE: partial splenic embolization

EIS was successfully performed at our gastroenterology department. On day 30 after EIS, PSE was performed using eight 60-mm and six 100-mm C-stopper coils. Following the procedure, an embolization rate of 48% was confirmed by contrast-enhanced CT (Figure 2). On day 20 after PSE, the platelet count increased to 16.1 (x10,000/µL), and 28 days after PSE, TAR was performed. The platelet count on the day of surgery was 10.1 (x10,000/µL) (Figure 3).

 After platelet elevation was confirmed, surgery was performed. Under general anesthesia, a guide wire was placed from the left femoral artery in the descending thoracic aorta under TEE guidance. An 8-mm artificial graft was then anastomosed to the left axillary artery. After full heparinization cardiopulmonary bypass (CPB) was established and cooling was started. Cardiac arrest was obtained by retrograde coronary perfusion as complete circulatory arrest at a tympanic membrane temperature of 25°C. The ascending aorta was transected just before the left common carotid artery. Selective cerebral perfusion was initiated from the brachiocephalic artery, left common carotid artery, and left axillary artery. The distal anastomosis position was decided to be just before the left common carotid artery. An open stent graft (33 mm x 120 mm; Lifeline Co. Ltd., Japan) was guided into the aneurysm with a guide wire and deployed while checking the depth through TEE. Triplex advanced (28 mm, 4 branches) was anastomosed with two layers of sutures. Side-branch blood supply was initiated, and the left common carotid and brachiocephalic arteries were anastomosed with the artificial graft. The proximal side was transected at 1 cm above the sinotubular junction (STJ) and anastomosed with two layers of sutures. After the artificial grafts were anastomosed to each other, the aortic cross-clamp was released. Then, the artificial graft, which was anastomosed to the left axillary artery, was guided into the mediastinum. End-to-end anastomosis was performed with the side branch of the main artificial graft. CPB was withdrawn, and after confirming hemostasis, the wound was closed. The total CPB, aortic cross-clamping, selective cerebral perfusion, and lower extremity circulatory arrest times were 204, 161, 134, and 36 minutes, respectively. Transfusions consisted of 10 units of concentrated red blood cells, 12 units of fresh frozen plasma, and 20 units of platelets.

**Figure 4:**
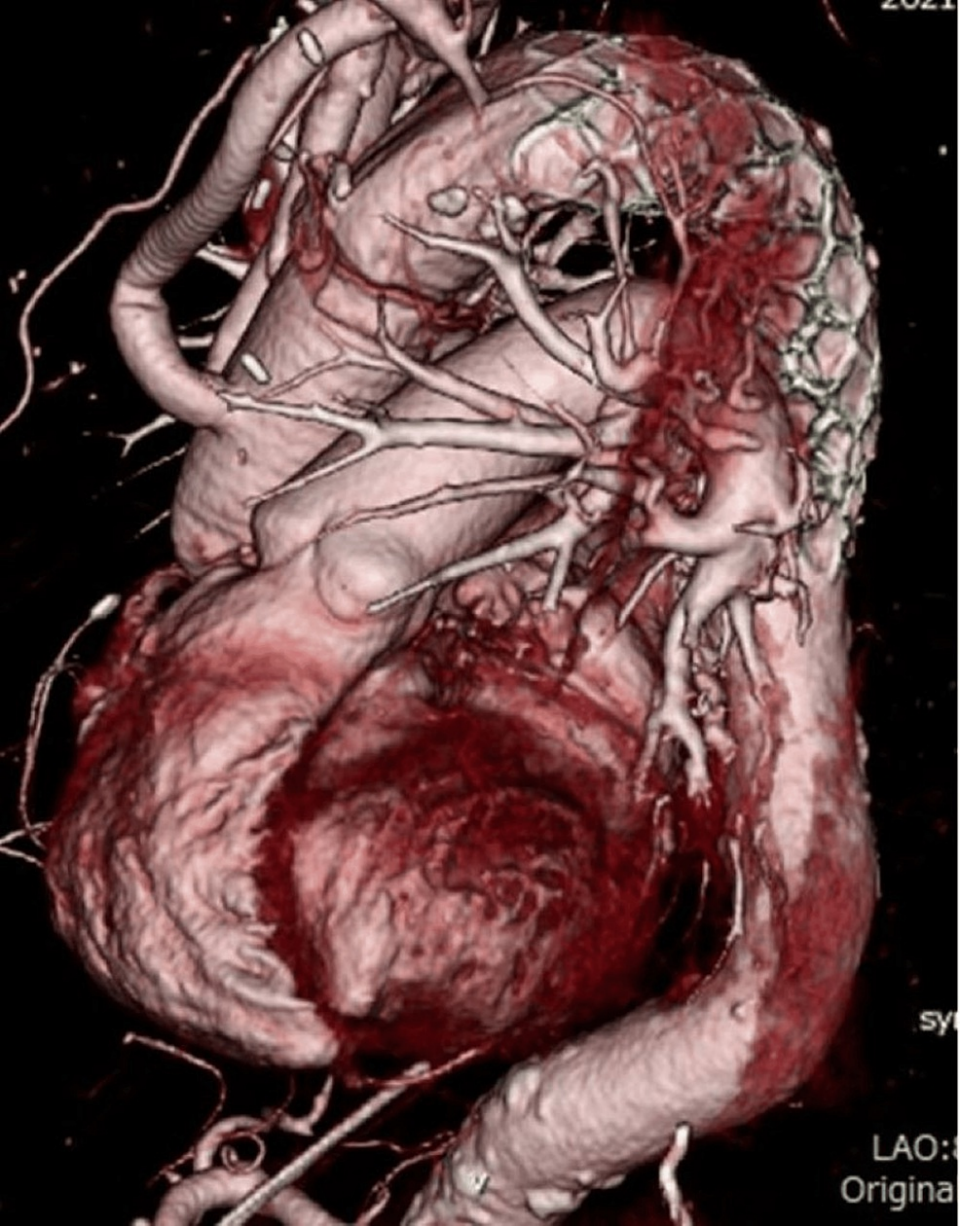
Postoperative computed tomography images No anastomotic abnormality was observed.

The patient was weaned from mechanical ventilation the day after surgery and was discharged from the ICU on the sixth postoperative day. She was discharged from the hospital on postoperative day 17 with good progress and without gastrointestinal bleeding, excessive drainage of blood, progressive anemia, mediastinitis, or other adverse events. Postoperative contrast-enhanced CT showed no anastomotic abnormalities (Figure 4). Platelets were not transfused after surgery, and the platelet count remained between 6.0 and 8.0 (x10,000/μL). There were no abnormal liver enzyme values. Two years after surgery, no complications have been noted.

## Discussion

There are approximately 400,000-500,000 LC patients in Japan. LC has a poor prognosis that is complicated by poor nutrition, abnormal coagulation, increased risk for infection, and renal dysfunction. The Child-Pugh classification is widely used to evaluate the risk of surgery while considering the complications of LC. The surgical outcome of CPB in patients with Child-Pugh classification A is reportedly 0%-10% while its complication rate is 25%-100% [2-8]. Some reports have used the MELD score to assess liver function and examine the surgical outcomes. The MELD score is a reliable indicator of short-term survival in patients with end-stage liver disease and is used to prioritize organ allocation for liver transplantation [9]. Although there are no established guidelines for using the MELD score in cardiac surgery, Thielmann et al. reported that a MELD score of ≥13.5 points indicated a high risk of hospital mortality [10]. Pathare et al. classified heart surgery cases according to the MELD score and reported a 31.2% mortality rate for patients with a MELD score of ≥20 points [11].

Complications of heart surgery for LC include bleeding associated with abnormal coagulation. In our patient, the platelet count was 9.3 (×10, 000/μL) at presentation; hence, it was suspected that hemostasis would be difficult to achieve. Therefore, preoperative PSE was performed to improve the platelet count. Thrombocytopenia in patients with LC is caused by splenomegaly associated with portal hypertension. PSE is an established treatment for splenomegaly [1]​​​​ and has evolved as a countermeasure against pancytopenia through a reduction in portal pressure in portal hypertension and other diseases aside from LC. PSE involves embolizing the spleen, which is the main blood supply route to the portal vein, thus reducing blood flow. Therefore, a synergistic effect is expected when PSE is performed in combination with direct treatment of esophageal varices. PSE combined with endoscopic esophageal variceal ligation for esophageal varices reportedly reduces variceal recurrence and bleeding compared to treatment of esophageal varices alone [12,13].

The pathophysiology of esophageal varices for which TEE should not be performed includes an RC sign of ≥1 [14].^ ^In our patient, the RC sign was 0, but form F2-3 and telangiectasia were observed. Gastroenterological examination revealed a high risk of bleeding from varicose veins during TEE; hence, EIS was initially performed, resulting in the embolization of varicose veins. As a result, TEE, guidewire verification, evaluation of cardiac function at the time of CPB withdrawal, and confirmation of the level of open stent graft placement were performed safely. Additionally, the platelet count increased to 15.3 (×10,000/μL) and 16.1 (×10,000/μL) on days 5 and 20 after PSE. Therefore, surgery was scheduled on the twenty-eighth day after PSE. On the day of operation, the platelet count decreased to 10.1 (×10, 000/μL), but this was still higher than the preoperative level.

Omori et al. reported that the rate of increase in the platelet count correlates with the rate of splenic embolization [15]. A PSE rate of >70% is associated with complications such as massive ascites, bacterial peritonitis, and splenic abscess, whereas a rate of <50% is associated with pancytopenia [16]. In our patient, the embolization rate was 48.3%, which may not have been sufficient. Additional embolization was initially considered but was not performed due to concerns regarding its complications.

No studies have explored the timing of heart surgery after PSE. However, Omori et al. reported an increase in platelet counts in the two-week to one-month period after PSE [15], and surgery within this period may be feasible.

## Conclusions

TAR was performed on a patient with LC. PSE and EIS were performed before TAR to manage the abnormal coagulation parameters and treat esophageal varices, respectively. This strategy resulted in a successful TAR without complications in a patient with LC.
